# Adenosine deaminase type 2 deficiency: From rare to common

**DOI:** 10.70962/jhi.20260015

**Published:** 2026-08-03

**Authors:** Marjon Wouters, Verena Kienapfel, Lisa Ehlers, Hasan Hashem, Ivona Aksentijevich, Isabelle Meyts

**Affiliations:** 1 Laboratory for Inborn Errors of Immunity, Microbiology Immunology and Transplantation, KU Leuven, Leuven, Belgium; 2 Department of Pediatric Respiratory Medicine, Immunology and Critical Care Medicine, Charité – Universitätsmedizin Berlin, Corporate Member of Freie Universität Berlin and Humboldt-Universität zu Berlin, Berlin, Germany; 3 Berlin Institute of Health at Charité – Universitätsmedizin Berlin, Berlin, Germany; 4 German Center for Child and Adolescent Health (DZKJ), Partner Site Berlin, Berlin, Germany; 5 Deutsches Rheuma-Forschungszentrum, An Institute of the Leibniz Association, Berlin, Germany; 6 Department of Pediatrics, Division of Pediatric Hematology-Oncology, King Hussein Cancer Center (KHCC), Amman, Jordan; 7 Pediatric Bone Marrow Transplantation and Stem Cell Therapy Service, King Hussein Cancer Center (KHCC), Amman, Jordan; 8 National Human Genome Research Institute, Bethesda, MD, USA; 9 Department of Pediatrics, Primary Immunodeficiencies, University Hospitals Leuven, Leuven, Belgium; 10 Jeffrey Modell Foundation Clinical Research Network and Diagnosis Center, New York, NY, USA

## Abstract

Deficiency of adenosine deaminase type 2 (DADA2) is an inborn error of immunity caused by biallelic pathogenic variants in the *ADA2* gene. DADA2 is characterized by a broad spectrum of clinical features, including inflammatory/vasculitic, hematological, and immunodeficient manifestations. Related to this complex clinical phenotype, which overlaps with other diseases, underdiagnosis of DADA2 can be suspected. Several pathophysiological mechanisms causing DADA2 have been described in recent years; however, a unifying pathomechanism explaining all DADA2 disease manifestations is still lacking. Although DADA2 is considered an autosomal recessive disorder, recently several variants in the ADA2 gene were described to cause DADA2 disease in a heterozygous state, via negative dominance. Data from PheWAS studies in large public databases support this observation. Based on these findings, the prevalence of ADA2-associated phenotypes might be much higher than the estimated 1 in 222,146 individuals. As a result, patients with these phenotypes are likely to be encountered by multiple medical disciplines.

## Teaser

Wouters et al. review the clinical and genetic landscape of DADA2, a multisystem inborn error of immunity. It examines expanding phenotypes, emerging pathophysiological mechanisms, treatment strategies, and newly described dominant-negative variants that challenge classical recessive inheritance—suggesting DADA2 is considerably more prevalent than previously recognized.

## Introduction

In 2014, two research groups reported for the first time a novel condition, defined as deficiency of adenosine deaminase type 2 (DADA2) in 27 patients from 14 kindreds mainly presenting with early-onset fevers, livedo racemosa, polyarteritis nodosa (PAN)-like vasculitis, and lacunar stroke. The cohort described by Zhou et al. displayed a broad phenotype, with features including strokes, immunodeficiency with prominent hypogammaglobulinemia, and lymphoproliferation, whereas the cohort described by Navon-Elkan et al. predominantly presented with PAN vasculitis (1, 2). The patient cohorts shared biallelic loss-of-function mutations in the *CECR1* gene (renamed to adenosine deaminase 2 [*ADA2*]), which correlated with a significantly reduced or absent ADA2 enzyme activity in the patients’ serum (1, 2). Already in the first papers, the wide variety of clinical presentations associated with DADA2 was striking. However, with over 600 DADA2 patients published to date worldwide, the phenotype has expanded (3, 4, 5, 6). Based on aggregate allelic frequency of deleterious variants in the Genome Aggregation Database and functional data on the enzymatic activity of these variants, in which 25% residual activity is used as the threshold, Jee et al. estimated a general incidence of 1 in 222,164 individuals corresponding to a carrier frequency of 1 in 236 individuals (7). Here, we provide a state of the art review on DADA2 as well as a recently proposed viewpoint on the dominant-negative effect of ADA2 variants (8).

## The ever-expanding phenotype of DADA2

**Figure 1.**
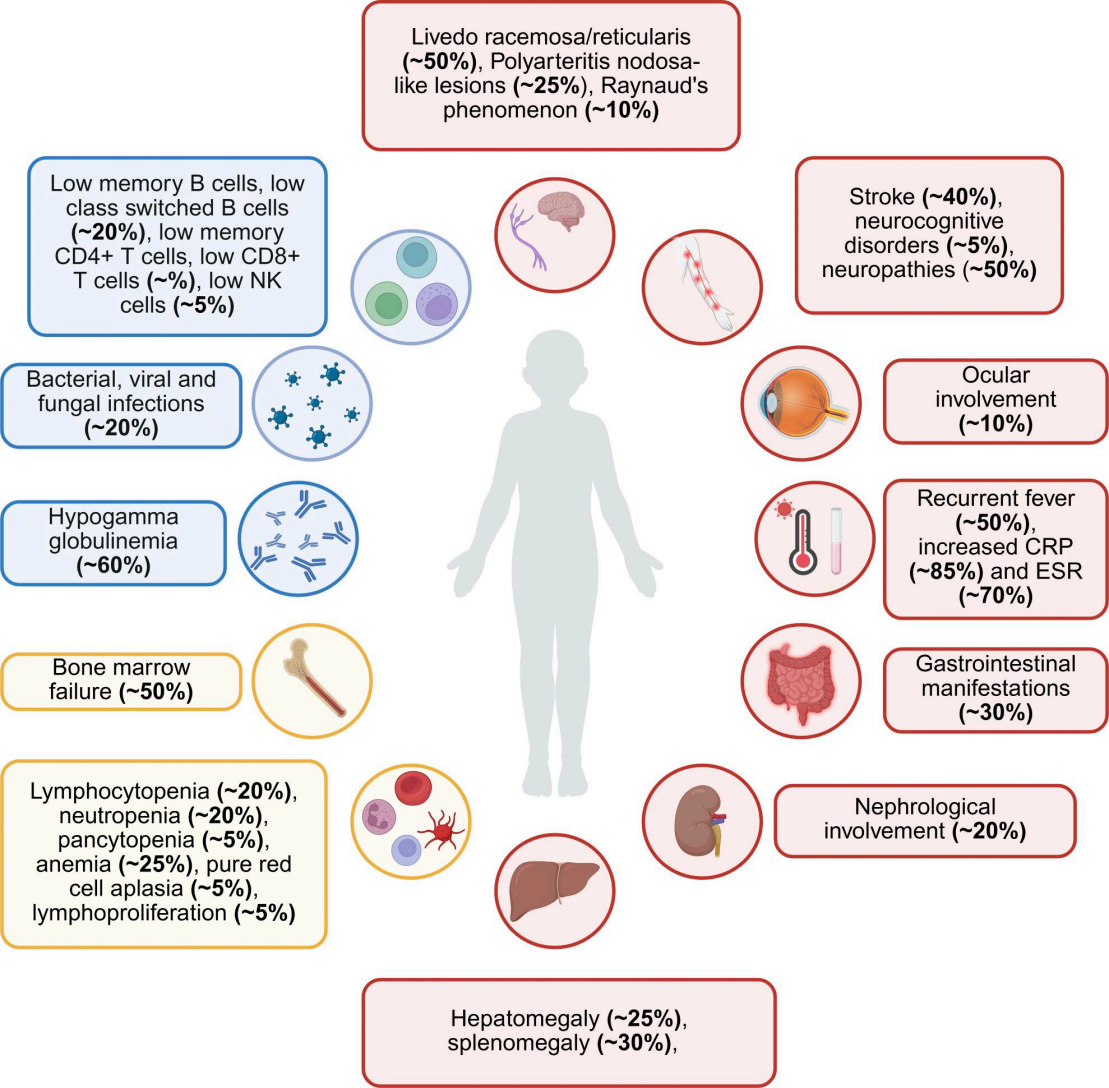
**An overview of DADA2 clinical phenotypes including the frequency of the respective phenotypes, as reported by Maccora et al., Lee et al., Dzhus et al., Barron et al., Li et al., Sharma et al., Rama et al., and Pulvirenti et al. (12, 13, 14, 15, 16, 17, 18, 19).** Colors denote the major different clinical phenotypes: red indicates the inflammatory/vasculitic phenotype, yellow indicates the hematological phenotype, and blue indicates the immunodeficient phenotype, acknowledging significant phenotypical overlap.

Overall, four predominant clinical phenotypes can be discriminated: (1) inflammatory/vasculitic, (2) hematologic, (3) immunodeficient, and (4) presymptomatic (patients harboring biallelic pathogenic variants in ADA2, yet currently asymptomatic) (9). However, overlapping phenotypes can be present in a single patient (9, 10, 11). An overview of the different disease manifestations in DADA2 is depicted in Fig. 1.

Around 70% of DADA2 patients present with an inflammatory/vasculitic phenotype affecting the skin, brain, as well as several end-organs such as the gastrointestinal (GI) tract, liver, and kidney (12). Skin manifestations can range from livedo racemosa/reticularis, PNA-like lesions, Raynaud’s phenomenon with constitutional symptoms, including recurrent fevers, and elevated inflammatory markers, including C-reactive protein and erythrocyte sedimentation rate (4, 11, 13, 14, 15). The most important neurological manifestations stem from early-onset, often recurrent strokes, with ischemic strokes occurring more frequently than hemorrhagic strokes (1, 2, 16, 17). Neurological features include headaches, neuropathies, neurocognitive disorders, and ocular involvement (18). GI tract involvement occurs in ∼30% of DADA2 patients and may reflect vasculitis with ischemia and/or inflammation. Clinical manifestations include diarrhea and vomiting, GI bleeding and bowel ischemia with perforation or necrosis, or ulcerative disease (1, 10, 12, 15, 19, 20, 21, 22). Other end-organ manifestations involve renal hypertension, hepatomegaly, portal hypertension, splenomegaly, regenerative and focal nodular hyperplasia of the liver, as well as cardiac involvement (1, 4, 13, 19, 20, 23, 24, 25, 26).

Besides the inflammatory phenotype, patients can present with a hematological disease, which can be the only presenting feature or alongside additional phenotypic features of DADA2. Cytopenias typically present early in life and include lymphopenia, neutropenia, pancytopenia, anemia, and pure red cell aplasia (PRCA) (1, 4, 15, 19, 27, 28, 29). The hematological phenotype is either the result of autoimmunity or of bone marrow (BM) failure (27, 30). Zoccolillo et al. showed that the BM of DADA2 patients contained decreased numbers of hematopoietic stem and progenitor cells (HSPCs) (31). Miano et al. observed an inflammatory BM environment in DADA2 patients, and BM progenitor cells showed decreased growth potential in an *in vitro* setting (32). This was corroborated by Bulté and colleagues, who identified a significant decrease in multilineage progenitor cells, with reduced differentiation potential (33). Additionally, DADA2 mesenchymal stromal cells showed reduced proliferation and impaired clonogenicity. In contrast, the number of BM cytotoxic CD8^+^ and terminally differentiated effector memory CD8^+^ T cells was found to be higher, likely contributing to the hematological phenotype (33).

A common immunological manifestation in DADA2 patients is hypogammaglobulinemia with ensuing recurrent bacterial infections (10). In fact, in a cohort of 181 patients diagnosed with common variable immunodeficiency, ∼6% were found to have DADA2 (22). DADA2 patients were found to have relatively increased numbers of transitional and naïve B cells and impaired immunoglobulin class switching (4, 22, 34, 35). The developmental arrest was observed within the BM B cell compartment, leading to impaired proliferation and terminal maturation of B cells (35, 36).

Furthermore, the numbers of memory CD4^+^ and CD8^+^ T cells and NK cells in the peripheral blood of patients were severely reduced (36). Peripheral blood T cells from patients displayed exhausted and senescent phenotypes, suggesting a broad dysregulation in T cell signaling. ADA2 deficiency also affects the function of T CD39^+^ regulatory cells and T follicular helper cells, further contributing to the reduced B cell function (35, 36). Immune repertoire sequencing of peripheral blood lymphocytes showed restricted T cell and B cell repertoires (37), which may explain why some patients are prone to develop lymphoproliferative disorders (3, 38, 39, 40, 41, 42). Although bacterial infections are the most common, viral infections and fungal infections have been described as well, which further supports that the defects are not only limited to the B cell compartment (6, 22, 28, 43, 44). Moreover, there appears to be a distinctive susceptibility to prolonged or severe disease following infection with Epstein-Barr virus, which can be linked to chronic and malignant lymphoproliferation (45, 46, 47, 48). Hemophagocytic lymphohistiocytosis has been reported in DADA2 patients and can be life-threatening (4, 44). In addition to B and T cell defects, immunophenotyping of NK cells from 11 DADA2 patients showed decreased NK cell frequencies and differentiation, with NK cells demonstrating a significantly reduced cytotoxicity against K562 target cells compared to healthy donor (HD) NK cells. Moreover, DADA2 NK cells showed a trend toward a more exhausted phenotype, which fits the lymphocyte exhaustion (49).

These findings revealed that immunological abnormalities can occur across all phenotypic presentations of DADA2 and suggest that ADA2 may have a function in the differentiation of hematopoietic cells.

## Mimics of ADA2 deficiency

Given the complex clinical phenotype, spanning over multiple medical disciplines, and overlapping with other diseases, DADA2 is likely underdiagnosed. For instance, some cases of Sneddon disease, characterized by ischemic stroke, livedo racemose, and antiphospholipid antibodies, have been diagnosed with DADA2. However, ADA2 deficiency only explains a small fraction of cases with Sneddon syndrome (50, 51, 52). Within the spectrum of autoinflammatory diseases, DADA2 can clinically mimic disorders such as Familial Mediterranean fever (FMF) and Behçet disease, given the frequent presentation of DADA2 with recurrent fever episodes, arthritis, and abdominal pain, especially in childhood (12, 21, 53, 54). In addition, some DADA2 patients have initially been diagnosed with autoimmune lymphoproliferative syndrome (ALPS), Diamond-Blackfan anemia (DBA), Multicentric Castleman’s disease, and Hodgkin lymphoma (3, 30, 40, 41, 42, 55). While hypogammaglobulinemia and lymphopenia may serve as helpful diagnostic clues, differentiation from ALPS relies on the broader and more distinct clinical spectrum of DADA2 (56, 57). For DBA, the absence of dysmorphic features and the presence of immunological abnormalities can point to DADA2 rather than DBA (56, 57, 58).

Due to recessive inheritance of DADA2 and its higher prevalence in founder populations, patients with molecularly confirmed DADA2 were reported to have monoallelic or biallelic FMF-associated mutations in the Mediterranean fever gene. Whether these variants amplify inflammatory phenotypes of DADA2 remains unclear (59). In a case of ALPS, Ahn et al. described a sibling pair with compound heterozygous missense variants in ADA2 as well as a heterozygous mutation in FAS (60). Such findings are not unusual and do not only highlight the need to investigate for additional genetic contributors in patients from founder populations but also emphasize the potential contribution of variants in disease-modifying genes to overlapping phenotypes, further complicating diagnosis.

## Treatment options for patients with DADA2

### TNF inhibition and others

TNF is a key cytokine in the pathogenesis of DADA2. Several studies have shown detectable levels of TNF in peripheral blood and biopsy tissues from patients (1, 61, 62, 63). Consequently, TNF inhibitors (TNFis) have been shown to attenuate the inflammatory and vasculitic symptoms. The clinical efficacy of TNFi for the inflammatory features of DADA2 was noted in earlier studies (2, 23), and more recent studies showed a clear reduction in the risk for stroke after initiation of TNFi (64, 65). TNF blockade is therefore the therapy of choice for vasculitis and inflammatory phenotypes (9). TNF receptor fusion protein etanercept and monoclonal anti-TNF antibodies adalimumab and infliximab are commonly used TNFis. However, TNF blockade is not effective in most DADA2 patients with severe hematologic phenotypes (9, 30). Moreover, TNFi agents may be accompanied by side effects such as infection and a potentially higher risk of malignancies (66). This is especially relevant in patients with severe hematological manifestations (e.g., BM failure, PRCA, and severe neutropenia). Importantly, antidrug antibodies can develop against TNFis, leading to flares of inflammation and stroke even though dose escalation or addition of immunosuppressives such as methotrexate have been able to reduce TNFi immunogenicity (67). For patients who have no access to these biologicals, thalidomide can be an alternative (11).

Collective experience suggests that the use of other immunosuppressive agents, including steroids, but also anti-IL-6, is largely ineffective for most patients (11, 13, 23, 68). Although these agents can be used to control inflammation in the acute phase of the disease, they often show a modest or short-term response. Cyclosporin treatment has been proposed to bridge to transplantation, with variable success (69). Moreover, inflammatory flares are common upon tapering (1, 2, 70). JAK-STAT inhibitors have been used in some DADA2 patients with a prominent interferon gene expression signature, although their efficacy in TNFi-refractory cases remains unclear (71). Type I IFNs have been shown to mediate hematopoietic suppression in aplastic anemia (72). Chen et al. recently demonstrated via bulk RNA sequencing the upregulation of both TNFα and type I IFN signaling in PBMCs of DADA2 patients compared to healthy controls (73). However, no differences were detected between symptomatic and asymptomatic DADA2 patients (73). These data suggest that multiple signaling pathways may contribute to BM failure in DADA2. Further studies are needed to assess the therapeutic value of JAK-STAT inhibitors in this disease. Indeed, combination therapy may be needed to control refractory disease.

Unfortunately, a lethality rate of 8–17% is linked to DADA2, due to vascular complications including cerebrovascular events and bowel ischemia as well as infection in the context of BM failure or due to uncontrolled inflammation (4, 10, 13, 28, 74). However, these data may not fully reflect outcomes in the era of TNFi, highlighting the need for updated evidence to reassess lethality.

### Hematopoietic cell transplantation for DADA2

As DADA2-related immunodeficiency and BMF are often refractory to TNFi, these patients require hematopoietic stem cell transplantation (HSCT), which is currently the only available curative treatment option (25). Hashem et al. have collated the multicentric world experience of HSCT in 30 patients of DADA2 who underwent a total of 38 HSCTs (25, 75). Indications for HSCT were BM failure, autoimmune cytopenias, lymphoproliferation, and immunodeficiency phenotypes. Overall survival in this HSCT cohort at 2 years was 97%, and HSCT resolved all the hematological phenotypes in all patients. Plasma ADA2 activity was restored to normal as early as 2 wk post-HSCT (76). Moreover, data showed that HSCT improved vasculitis. When considering HSCT in DADA2 patients, it is essential to design preparative regimens to address the specific vulnerabilities, particularly endothelial dysfunction and liver toxicity. It is also essential to mitigate the risk of graft rejection while minimizing toxicities and graft-versus-host disease. This can likely be achieved by optimizing immunoablation using serotherapy in DADA2 patients (26). A new international study is underway and may help define the optimal transplant conditioning regimen.

### Future therapeutic perspectives

Gene therapy using autologous HSPCs could be the life-saving breakthrough for DADA2 patients. Zoccolillo et al. performed lentiviral (LV) delivery of wild-type (WT) ADA2 into HSPCs from HDs, yielding expression of a functional protein (31). Differentiation and engraftment of human and mouse HSPCs *in vivo* was not affected by supranormal ADA2 protein expression after LV transduction. The LV-ADA2 induced stable ADA2 expression and corrected the enzymatic defect in HSPCs from DADA2 patients. Moreover, LV delivery of functional ADA2 into DADA2 macrophages restored normal expression levels of inflammatory cytokines, providing proof of principle for the development of gene therapy (31). Similar findings were obtained independently by Hong et al., who also corrected HSPCs from a DADA2 patient with PRCA, highlighting the potential for curing these patients as well (31, 77, 78). More recently, Rigamonti and colleagues demonstrated restoration of ADA2 protein expression and enzymatic activity in DADA2 patient-derived HSPCs *in vitro*, and described successful sustained long-term engraftment and multilineage reconstitution in a mouse model, at levels comparable to HDs (79). These findings suggest that LV-mediated ADA2 gene therapy can be an alternative to allogeneic HSCT (79). Moreover, gene-editing therapies will be another option in the future for treating patients who are carriers of common pathogenic mutations.

Like for other genes with multiple pathogenic variants, modulator therapy, aiming to correct ADA2 levels to a sufficient level, may form an alternative treatment option. Proof of principle was obtained *in vitro* and *in vivo* with the use of hydroxychloroquine, which blocked lysosomal ADA2 degradation, resulting in a partial restoration of the ADA2 protein expression and reversal of neutropenia in a patient with long-standing agranulocytosis (80). Finally, pegylated ADA2 enzyme replacement therapy may improve disease symptoms in patients who lack access to TNFi or are unsuitable candidates for HCST (81).

### ADA2: The gene and protein

**Figure 2.**
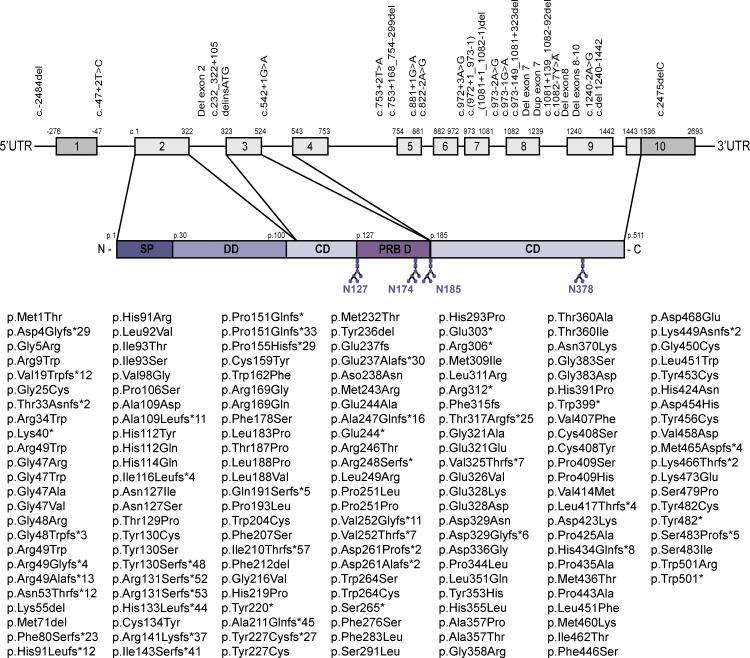
**Schematic representation of the *ADA2* gene and ADA2 protein with disease-associated mutations adapted from Dzhus et al. (15).** SP, signal peptide; DD, dimerization domain; CD, catalytic domain; PRBD, putative receptor–binding domain.

The *ADA2* gene encodes a 511-amino acid protein that comprises four domains, each embodying several characteristics of the protein as depicted in Fig. 2 (82). ADA2 secretion is facilitated by the N-terminal signal peptide. The dimerization domain mediates the interaction between two ADA2 monomers by forming a hydrophobic area. The catalytic domain accounts for the largest portion of the protein, mediating both the enzymatic activity and dimer formation. Lastly, within the catalytic domain, there is a putative receptor-binding domain, with hitherto unknown function (82).

Along with the expansion of the clinical phenotype, the pool of pathogenic variants in *ADA2* causing the disease has grown tremendously. A total of 116 pathogenic and likely pathogenic variants are listed in the Infevers database and are spread across all protein domains (Fig. 2) (83). While the majority of *ADA2* variants associated with DADA2 clinical disease showed a residual ADA2 enzyme activity <25% in a HEK293T overexpression system, some DADA2-associated variants had a residual activity up to 75% of the WT protein (7).

Like many other secreted proteins, ADA2 undergoes N-glycosylation in the endoplasmic reticulum (ER), which is important for proper folding and enzymatic activity of the ADA2 protein. There are four N-glycosylation sites identified at p.N127, p.N174, p.N185, and p.N378, all of which have been shown to be glycosylated in the HEK293T overexpression system (84, 85). However, mutations affecting glycosylation sites do not equally impair protein integrity; while loss of glycosylation at p.N127, p.N174, and p.N378 results in ER retention, p.N185 retains normal protein secretion and function (85). Independent of their location across the *ADA2* gene, pathogenic variants have been associated with ER retention and an increased ER stress response (2, 73, 85, 86). Recently, our group has identified an intracellular glycoform of ADA2 in human monocyte-derived macrophages (HMDM) of HDs (87). This glycoform has reduced molecular weight due to trimming by alpha-mannosidases, and it is not expressed in DADA2 patient-derived HMDMs. We propose that this low molecular-weight glycoform represents the biological isoform of the lysosomal form of ADA2 (87).

### Genotype–phenotype correlation

Navon-Elkan et al. reported the predominant phenotype of childhood-onset PAN-like disease in the Georgian Jewish population associated with the p.G47R founder variant (2). The same variant is also commonly found in India and Turkey, and across these cohorts, DADA2 cases with the p.G47R variant exhibited predominantly inflammatory vasculopathy (14, 55). The early series of DADA2-associated PRCA and BM failure (BMF) were linked to a different set of pathogenic ADA2 variants (29, 55, 88). Ozen and colleagues proposed that variants in the dimerization domain of ADA2 (i.e., p.G47R/A) are associated with the vasculopathy phenotype, while variants in the catalytic domain are linked to a hematological phenotype (55). Lee et al. tested the effect of pathogenic variants in HEK293T cells (30). From this analysis, it appeared that variants associated with the vasculopathy phenotype tended to display detectable residual ADA2 enzyme activity *in vitro*. In contrast, the PRCA and BMF phenotypes are associated with loss-of-function (LOF) variants (i.e., nonsense and frameshift) and missense variants with minimal or null residual activity.

It is worth noting that specific missense variants with very low enzymatic activity, such as p.R169Q that is frequent in Northern Europe, are linked to a broad spectrum of clinical features, including vasculopathy, BMF, and immunodeficiency. Disease manifestations depend at least in part on whether this variant is inherited in homozygosity or together with another milder variant (28, 75, 89). Intrafamilial disease expressivity is also variable, with incomplete penetrance (28, 63, 89). One potential explanation relates to the effect of this variant on ADA2 dimerization and its secretion. The amino acid Arg169 supports the structural integrity of the ADA2 dimerization interface and is critical for the enzyme’s stability and function by electrostatic or hydrogen-bonding interactions (90). The glutamine substitution, which removes the charged side chains, could weaken monomer–monomer interactions, resulting in protein misfolding and failure of proper secretion.

### Molecular characteristics and differences with ADA1

Both ADA1 and ADA2 are adenosine deaminases, responsible for the deamination of adenosine and deoxyadenosine (dA) (91, 92, 93). However, the functions of ADA1 and ADA2 are not redundant, as exemplified by deficiency of ADA1, which results in ADA-SCID, due to the intracellular accumulation of toxic purine metabolites (94). In contrast to ADA1, ADA2 forms dimers, carries a signal peptide, and is secreted by monocytes, macrophages, and dendritic cells. In line with these findings, ADA2 is suggested to function as the extracellular adenosine deaminase, while ADA1 is considered the intracellular adenosine deaminase (10). Surprisingly, in dendritic cells, ADA2 was responsible for most of the intracellular adenosine deaminase activity (95). In terms of affinity for its substrates, ADA1 has a 50-fold higher affinity for adenosine compared to ADA2. Besides, ADA2 exhibits reduced activity toward dA compared to adenosine (96, 97, 98) and has a slightly reduced optimal pH at 6.5 compared to that of ADA1 (97, 99). Interestingly, even before its role in diagnosing ADA2 deficiency, ADA2 levels were assessed for diagnostics in plasma or pleural effusions in the context of inflammation, infection, or cancer (5, 100, 101, 102, 103, 104, 105). Together, these distinct enzymatic properties, expression profiles, and presentations of deficiency point to unique physiological roles for both ADA1 and ADA2.

### Insights into the physiological role of ADA2 and hints toward the pathophysiology of DADA2

**Figure 3.**
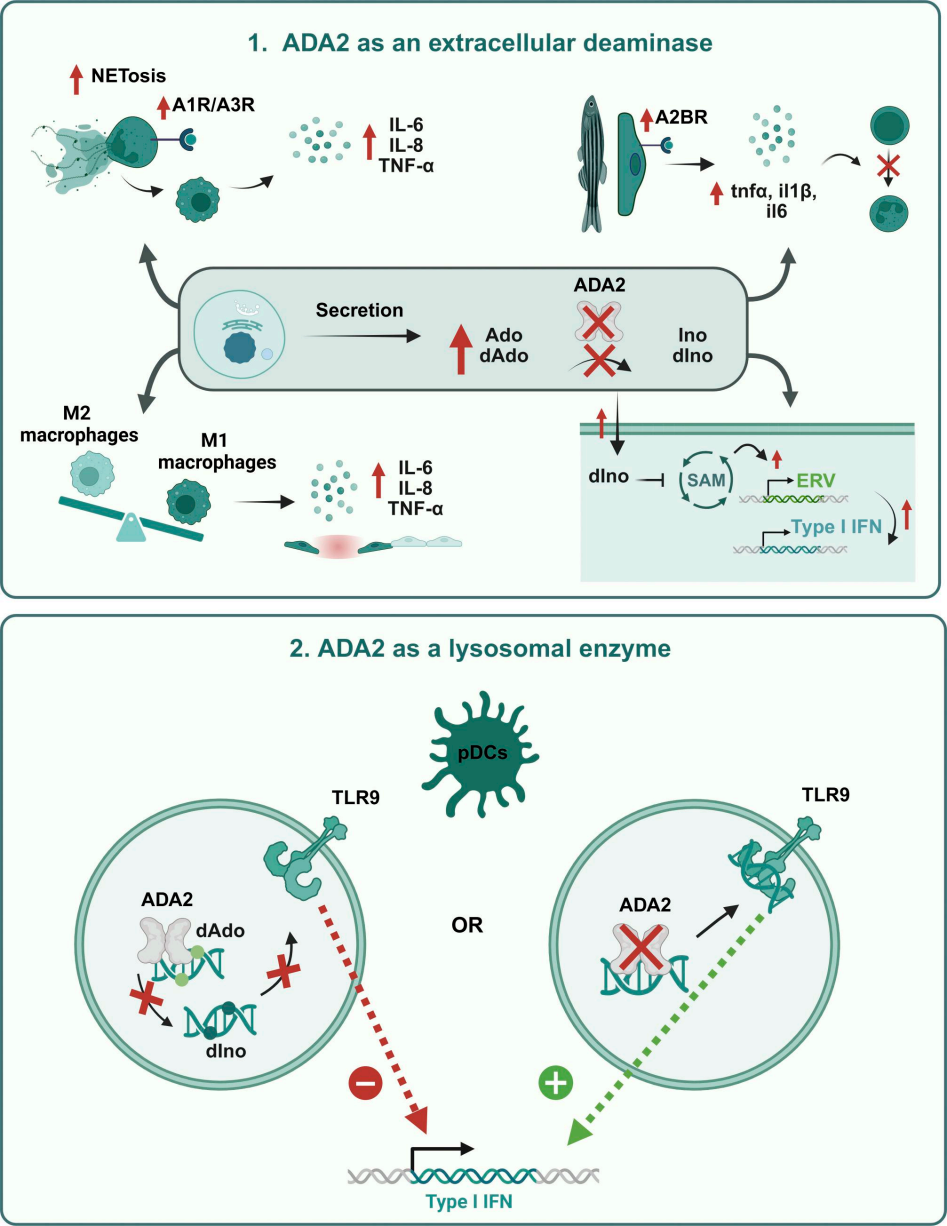
**Proposed DADA2 pathomechanisms focusing on ADA2 as an extracellular adenosine deaminase or as a lysosomal protein (1, 97, 111, 112, 113, 114, 115, 116).** Ado, adenosine; dAdo, deoxyadenosine; dIno, deoxyinosine; ERV, endogenous retroviral elements; HSC, hematopoietic stem cell; HUVECs, human umbilical vein endothelial cells; Ino, inosine; NET, neutrophil extracellular traps; SAM, s-adenosyl methionine.

There is an ongoing quest for the understanding the pathophysiology of this puzzling condition (Fig. 3). Although ADA2 exists in lower species, the lack of its homolog in mice has hindered our understanding of ADA2 functions. The diagnostic gold standard rests on the detection of biallelic deleterious variants in the *ADA2* gene and the finding of reduced serum or plasma ADA2 activity (9). Several methods are available to measure ADA2 enzyme activity in serum, plasma, or dried-blood spots, including spectrophotometric/colorimetric methods measuring the byproduct ammonium, or high-performance liquid chromatography, or liquid chromatography-tandem mass spectrometry measuring inosine (1, 5, 11, 55, 106, 107, 108). However, when DADA2 patient variants are tested in a HEK293T transient overexpression system, the residual activity is quite variable (7, 30, 73).

### ADA2 as an extracellular protein

A plethora of data has indicated that ADA2 plays different roles. In the early 2000s, it was suggested that ADA2 may have growth factor functions based on its homology to fly growth factors and role in *Drosophila* development (82, 109, 110, 111). Moreover, *in vitro* studies have demonstrated that ADA2 binds to different subsets of immune cells than ADA1, specifically CD26-negative immune cells, indicating a role in maintaining immune synapses (112). Next, Zavialov et al. demonstrated that ADA2 may regulate the interactions between myeloid and adaptive immune cells. *In vitro* studies with recombinant ADA2 showed that ADA2 acts as a co-stimulatory factor in CD4^+^ T cell proliferation by enhancing monocytes–CD4^+^ T interactions, and supports monocytes differentiation into macrophages (95). In the initial description of DADA2, the primary patients’ monocytes showed a preference to differentiate into M1 pro-inflammatory macrophages while poorly developing into anti-inflammatory M2 macrophages (1, 61). This was further substantiated by Zhu et al., who reported that ADA2 promotes macrophage differentiation toward M2 macrophages in glioblastoma multiforme (113). Therefore, it was hypothesized that ADA2 acts as a switch in macrophage differentiation, where absence of the protein results in a shift toward M1 macrophages, leading to endothelial inflammation and the vasculitic/inflammatory phenotype. This switch might be cell specific, as the recombinant human ADA2 can promote a pro-inflammatory and pro-fibrotic liver phenotype in HMDM*.* This effect was dependent on the adenosine deaminase activity (114).

The adenosine deaminase function of the ADA2 protein is thought to play a role in regulating extracellular purine metabolism. Carmona-Riviera and colleagues proposed that adenosine mediates neutrophil extracellular trap formation (NETosis) via specific adenosine receptors, A1 and A3, contributing to the DADA2 pathophysiology (115). They observed increased adenosine levels in the plasma of DADA2 patients, and higher numbers of low-density granulocytes (LDGs) prone to spontaneous NETosis (115). Interestingly, increased circulating LDGs were found in unaffected family members who carry monoallelic *ADA2* mutations. Co-culture of NETs isolated from DADA2 patients with HD M1 macrophages caused an increase in TNF-α production (115). This study hypothesized that the absence of ADA2 in myeloid cells leads to adenosine accumulation, triggering spontaneous NET formation, and subsequently induces TNF-α production by M1 macrophages (115). ADA2-deficient myeloid cells produce more TNF and other inflammatory cytokines, resulting in systemic inflammation, endothelial cell damage, and vasculopathy (116). Hence, vascular inflammation is alleviated with TNFi (61).

In addition, DADA2 immune cells exhibit both type I and type II interferon gene expression signatures (62, 73, 117, 118, 119, 120). Importantly, upregulated type I and type II IFN signaling was also observed in patients receiving TNF-α inhibitors despite the resolution of the inflammatory state (62, 120). Dhanwani et al. have suggested that the extracellular deaminase function of ADA2 plays a role in regulating DNA methylation (121). In the absence of extracellular ADA2, the accumulation of intracellular deoxyinosine (dI) inhibits s-adenosyl-methionine synthase, promoting the expression of endogenous retroviral elements and activation of double-stranded RNA MAVS-sensing pathways. Ultimately, this process results in an upregulation of the type I IFN response and the subsequent inflammatory phenotype in DADA2 (121). However, a major limitation of this study is that it is conducted *in vitro* with overexpressed recombinant proteins, which may not accurately reflect the physiological role of the endogenous protein.

The described pathomechanisms connect deficient extracellular adenosine deaminase activity to the inflammatory phenotype of DADA2 and do not explain the hematological and immunological manifestations. There are two paralogs of *CECR1* in zebrafish, *cecr1a* and *cecr1b*. Brix and colleagues made use of this zebrafish model to link deficient ADA2 activity to the hematological phenotype of DADA2 (122). Here, both knockdown and knockout of the ADA2 zebrafish orthologue *cecr1b* resulted in a DADA2-like phenotype of inflammation and neutropenia. LOF mutations in *cecr1b* initiated hyperactivated adenosine-mediated A2BR signaling, resulting in endothelial inflammation caused by an increase in proinflammatory cytokines including *tnfα* as well as vascular dysfunction. Consequently, dysregulated hemogenic endothelial cell specification can result in deficient HSPC emergence, expansion, and differentiation (122). Moreover, Inhibition of *tnfα* restored low HSPC numbers and reconstituted the neutrophil population in *cecr1b*-LOF embryos. This finding contrasts with the observation in DADA2 patients with a hematological phenotype, in whom TNFi is largely ineffective (122). A possible explanation could be that the long-standing inflammatory environment may already have caused substantial and irreversible hematopoietic damage, thereby limiting the therapeutic efficacy of TNF inhibition (122).

Zavialov et al. proposed that ADA2 regulates adenosine levels at sites of inflammation (90). In an inflammatory microenvironment, higher concentrations of adenosine and a lower pH may be more in line with the enzymatic optimum of ADA2. However, extracellular purine metabolism is important for controlling the ATP:adenosine ratio; while ATP promotes inflammation, adenosine exerts anti-inflammatory effects (123). Enzymes ectonucleoside triphosphate diphosphohydrolase 1 (CD39) and 5′-nucleotidase (CD73) convert ATP to AMP and subsequently to adenosine, fostering an anti-inflammatory response (124). Subsequently, the action of adenosine deaminases is pro-inflammatory as it augments the extracellular ATP:adenosine ratio by decreasing adenosine (124). This would mean that the absence of functional ADA2 should be anti-inflammatory since adenosine would accumulate. Although this hypothesis does not fit the pro-inflammatory context of ADA2 deficiency, the net adenosine function depends on which of its receptors are activated and on the specific cellular environment. Moreover, as mentioned previously, based on enzymatic characteristics, ecto-ADA1 should be able to compensate for the loss of functional ADA2 in the extracellular space (96).

### ADA2 as an intracellular lysosomal protein

Over the past year, two independent research groups have proposed a novel role for ADA2. In line with previous reports proposing that ADA2 is a lysosomal protein due to its colocalization with the lysosomal marker LAMP1 and the presence of mannose-6-phosphate–modified glycans on three of its glycosylation sites, recent findings have provided further support for the ADA2 intracellular localization (125). Dong et al. demonstrated that ADA2 colocalizes with the lysosomal marker LAMP2 in transfected HEK293T cells (126). As ADA2 exhibits peak enzymatic activity at pH 5, this localization is consistent with the acidic environment of the lysosome. Next, both Dong and Greiner-Tollersrud et al. reported DNA binding by ADA2, albeit with different specificities for different DNA ligands. Dong et al. assessed binding to various classes of CpG oligodeoxynucleotides (ODNs), finding the highest affinity for CpG ODN 2206 PTO—a linear, unstructured ODN (126). Greiner-Tollersrud et al. examined a range of nucleic acid structures and found that ADA2 has the highest affinity for complex, branched DNA forms such as hairpins containing both single- and double-stranded regions (127). Both groups also suggest that ADA2 has immunomodulatory functions related to TLR9 signaling, although through different mechanisms. Dong et al. proposed that ADA2 competes with TLR9 for CpG DNA binding in the endosomal/lysosomal compartment, and that its absence enhances pro-inflammatory signaling—evidenced by an upregulated inflammatory response in ADA2 knockdown plasmacytoid dendritic cells(pDCs) (126). Hence, this could explain the inflammatory phenotype of DADA2. Meanwhile, Greiner-Tollersrud et al. reported that ADA2 binds nucleic acids with greater affinity than free adenosine, and that is important for editing of DNA molecules by modifying dA to dI in the lysosomes (127). This enzymatic modification enhances TLR9 signaling, since dI is read as deoxyguanosine by the DNA sensing machinery. In this model, the absence of ADA2 results in reduced dI production, dampening TLR9 activation, and is contradictory to the inflammatory phenotype in DADA2 (127). However, a similar paradox has been observed with the function of the DNase II enzyme (128).

It has also been observed that plasma ADA2 has lower activity toward adenosine than dA, whereas it is more active on dA in DNA. A potential explanation is the difference in the pH environments, with an acidic pH in the lysosomes as opposed to a neutral pH in the plasma. The major discrepancies between these findings could include the use of different cellular systems (primarily pDCs by Dong et al. vs. primary human cells and reporter cell lines by Greiner-Tollersrud et al.) and the differences in experimental conditions (focus on early endosomes by Dong et al. vs. focus on lysosomes by Greiner-Tollersrud et al.). Further research is needed to solve or reconcile these apparent contradictions in the function of ADA2 and the pathophysiology of DADA2.

### DADA2 carriers: The case for autosomal dominant DADA2

DADA2 carriers are classified as individuals harboring monoallelic pathogenic variants in the *ADA2* gene (9). Although most ADA2 carriers are healthy and asymptomatic, several independent reports in the literature, including the very first description by Zhou and colleagues (1) that described susceptibility to late-onset strokes in two siblings, DADA2 carriers, documented overt phenotypes. An overview of these reports is presented in Table 1 (1, 106, 120, 129, 130, 131, 132, 133). While the phenotype of individuals who are heterozygous carriers of specific pathogenic variants is mainly characterized by vasculitic/inflammatory manifestations, hematological abnormalities such as anemia and immune dysregulation, including recurrent infections and common variable immunodeficiency, have also been observed. It is important to note that recessively inherited DADA2 can be linked to the presence of pathogenic copy number variants, such as small genomic deletions or duplications, which are not easily detected by Sanger sequencing or whole-exome sequencing. Thus, in some reported cases, the second mutation might have been missed. Nevertheless, if this were the case, it would likely result in strongly reduced serum ADA2 enzyme activity.

**Table 1.** Overview of reported diseased DADA2 carriers in literature

Study	Age at onset (years)	ADA2 genotype	Clinical phenotype	ADA2 activity	Treatment
Zhou et al. (1)	Adulthood	p.Y453C/WT	Small vessel ischemic stroke	Unknown	Unknown
Zhou et al. (1)	Adulthood	p.Y453C/WT	Hypertension, small vessel ischemic stroke	Unknown	Warfarin
Rama et al. (130)	5	p.G47R/WT	Recurrent fever, elevated CRP levels, cephalgia, arthralgia, myalgia, papular rash with pruritic and GI manifestations	Carrier range	Unknown
Nihira et al. (120)	10	p.E328K/WT	Fever, intracranial hemorrhage, cerebral infarction, livedo racemosa, splenomegaly, and renal infarction	Low	Infliximab and SCIG replacement
Nihira et al. (120)	12	p.E328K/WT	Diplopia, memory disturbances, livedo racemosa, and renal infarction	Low	Infliximab and SCIG replacement
Schnappauf et al. (106)	Adulthood	p.Y453C/WT	PAN with fever, cutaneous ulcers, and neuropathy	High carrier range	Unknown
Moi et al. (131)	Childhood	p.R169Q/WT	Arthritis, recurrent infections, and common variable immunodeficiency	Carrier range	IVIG replacement
Celikel et al. (134)	1.5	p.G47R/WT	Recurrent fever, elevated CRP levels, and anemia	Unknown	Etanercept
Izumo et al. (132)	9	p.F355L/p.Y453C	Elevated CRP levels, livedo, and multiple cerebral infarctions	Carrier range	Infliximab
Pulvirenti et al. (129)	12	p.Y453C/WT	Fever, skin rash, hypogammaglobulinemia, and lymphadenitis	Unknown	No treatment
Yilmaz et al. (133)	20	p.R49Afs*13/WT	Crohn’s disease	Carrier range	Surgery plan

The biochemical testing of blood samples identified a range of ADA2 activities in the patients and healthy individuals. Individuals with ADA2 activity in the carrier range, whether harboring monoallelic or biallelic variants in ADA2, are more challenging to diagnose clinically. Genotype segregation analysis is also critical for the genetic diagnosis. For example, one study reported a severely affected young patient carrying two ADA2 variants and with intermediate ADA2 enzymatic activity (132). One variant is classified as pathogenic (Y453C), while the other variant (F355L) is classified as a variant of unknown significance. However, because parental segregation was not performed in this and other reports, it remains unclear whether the ADA2 variants were inherited *in trans* or *in cis* on the same allele.

Recently, a report was published by the authors of this review, including 10 patients from 7 kindreds in which the individuals presented with DADA2 disease but carried only a single pathogenic variant (8). To study an underlying mechanism that could explain disease in these DADA2 carriers, *ADA2* mutants were transiently co-transfected with WT *ADA2* in HEK293T cells to model carrier status. Here, they observed that previously described pathogenic variants p.G47V, p.R169Q, p.H424N, and p.Y453C affected secretion of the WT ADA2 protein (8). Moreover, p.G47A, p.G47R, p.G47V, p.R169Q, p.E328K, p.H424N, and p.Y453C exert a dominant-negative effect on WT ADA2 enzymatic activity, both intracellularly as well as extracellularly (8). PheWAS data support the notion that, in addition to autosomal recessive (AR) DADA2, autosomal dominant (AD) DADA2 may exist. There is also the possibility of random monoallelic expression, which cannot be ruled out without formal testing (135, 136). Currently, the questions of why some DADA2 carriers are diseased while others are asymptomatic remains unresolved. Since the dominant-negative variants described in the study make up an important proportion of pathogenic variants described in DADA2 patients, this information is especially relevant for DADA2 patient kindreds (18). At present, the penetrance and clinical significance of dominant-negative and monoallelic variants in DADA2 are not well-defined, similar to other classically recessively inherited inborn errors of immunity (IEIs). Therefore, further research is needed to clarify their impact on IEI prevalence. Lastly, it could be questioned whether the data on the negative dominance of ADA2 variants on WT ADA2 might also be applicable to ADA2 variants with higher residual enzyme activity. *In vitro* HEK293T overexpression screening of ADA2 variants by Jee et al. revealed that although 91% of pathogenic ADA2 variants exhibit an enzymatic activity below 25%, several variants reported in patients displayed an enzymatic activity above 50% of WT ADA2 (7). This highlights the importance of not only functionally validating ADA2 variants in homozygous conditions but also performing testing in compound heterozygous conditions. Additional research is needed to decipher the impact of such dominant-negative variants at the cellular level. With regard to the treatment of heterozygous carriers, the findings in this study are specific for individuals with dominant-negative ADA2 variants (8). Although a specific immunological signature of DADA2 is lacking, it may be worthwhile to consider immunophenotyping and serum cytokine analysis, in addition to disease damage and severity of the manifestations. More research is needed to provide robust advice.

Incomplete penetrance and differences in disease expressivity are well-recognized features in the world of IEIs (137). For example, in classical AR DADA2, several asymptomatic individuals with molecularly confirmed DADA2 and absent ADA2 enzymatic activity were described (9, 14, 73). Of course, next to environmental triggers, a modifying allele in an undefined gene, microRNAs, epigenetic modifiers, or age-related occurrence of clonal hematopoiesis of indeterminate potential–associated somatic mutations in myeloid cells may account for the broad spectrum of observed immune phenotypes.

Taken together, these data suggest that the prevalence of DADA2 may be much higher than currently estimated 1 in 222,164 individuals (7). Given these prevalence estimates and the availability of effective therapies, inclusion of DADA2 in newborn screening programs may be warranted. Several pilot studies have demonstrated the feasibility of measuring ADA2 enzymatic activity in dried blood spots (106, 107). Confirmatory strategies could include plasma ADA2 enzymatic activity assays and genetic screening. However, broader implementation will require larger validation studies, standardized cutoffs, and evidence of population-level benefit.

## Conclusion

Human ADA2 deficiency is a heterogeneous IEI with a complex pathophysiology and manifests with a broad spectrum of clinical manifestations. TNFi are the recommended treatment for manifesting the vasculitic/inflammatory phenotype, whereas HSCT remains the only treatment option curing all DADA2 disease manifestations. In the future, genetically based therapies are anticipated to transform the treatment of DADA2. Elucidating the pathophysiological mechanisms in DADA2 is pivotal for understanding the full spectrum of ADA2 functions. Lastly, the observation that DADA2 carriers can develop clinical disease, and the proposed dominant-negative mechanism for at least a limited set of variants, suggests that DADA2 may be more common than IEIs are currently recognized and supports its inclusion in newborn screening programs.
